# Microfracture Technique for Chronic Unstable Osteochondral Defect of Knee: Case Report

**DOI:** 10.7759/cureus.13180

**Published:** 2021-02-06

**Authors:** Gur Aziz Singh Sidhu, Ilias Galanopoulos, Neil Ashwood, Francesco Bindi, Keith Hayward

**Affiliations:** 1 Trauma and Orthopaedics, University Hospitals of Derby and Burton, Burton, GBR; 2 Orthopaedics, University Hospitals of Derby and Burton, Burton, GBR

**Keywords:** osteochondral defect, microfracture, drilling, autografts, chondrocyte transplantation

## Abstract

Osteochondral fractures of the medial femoral condyle of the knee can be diagnostically and therapeutically challenging. Various techniques of osteochondral defect treatment include fixation, abrasion chondroplasty, drilling, microfracture, autografts, allografts and chondrocyte transplantation

A 37-year-old man presented with persistent left knee pain of about six months duration. Concomitant symptoms included swelling, several episodes of locking and clicking, and a sense of instability especially in walking downstairs. MRI scan revealed an unstable osteochondral lesion about 2 cm in diameter involving the medial femoral condyle. The patient underwent arthroscopic removal of the fragment and microfracturing of the defect on the medial femoral condyle. Postoperatively, he was treated with non-weight bearing for six weeks along with quadriceps strengthening and range of motion (ROM) exercises. The final outcome was good as the patient has returned to his previous activities.

Microfracture technique is quite effective with regard to the improvement of daily activities with a favorable impact on pain relief and overall satisfactory functional results.

## Introduction

The most common causes of osteochondral fragments in the knee joint, especially in patients younger than 40 years, are osteochondritis dissecans and a traumatic event. Various techniques of chondral or osteochondral defect treatment include fixation of the fragment back to its normal position, abrasion chondroplasty, drilling, microfracture, osteochondral autografts, osteochondral allografts, and chondrocyte transplantation [1,2].

The target of surgical treatment is to fill the cartilage defect with newly formed cartilaginous tissue or similar structures. The ultimate aim is to achieve normal knee function with establishment of integrity of this new regenerate with adjacent cartilage and subchondral bony structure [3].

The microfracture procedure is one of the marrow stimulation techniques [4], such as abrasion chondroplasty [5] or Pridie drilling [6], and has some inherent advantages. The microfracture technique is minimally invasive because it is performed arthroscopically through standard portals in most cases. In contrast to other methods such as abrasion chondroplasty, the subchondral bone plate is preserved, improving load-bearing characteristics after healing. In contrast to osteochondral, perichondral, periosteal, or chondral autograft procedures, the problem of harvest-site morbidity is excluded with microfracture technique. The technique has been elaborated by Steadman et al. [7] and applied mainly in young athletes and in young patients in general. Several clinical studies have shown improvement in function without significant deterioration over time [8]. However, animal studies have shown that the tissue formed after microfracture is fibrous in nature and not durable [9].

## Case presentation

**Figure 1:**
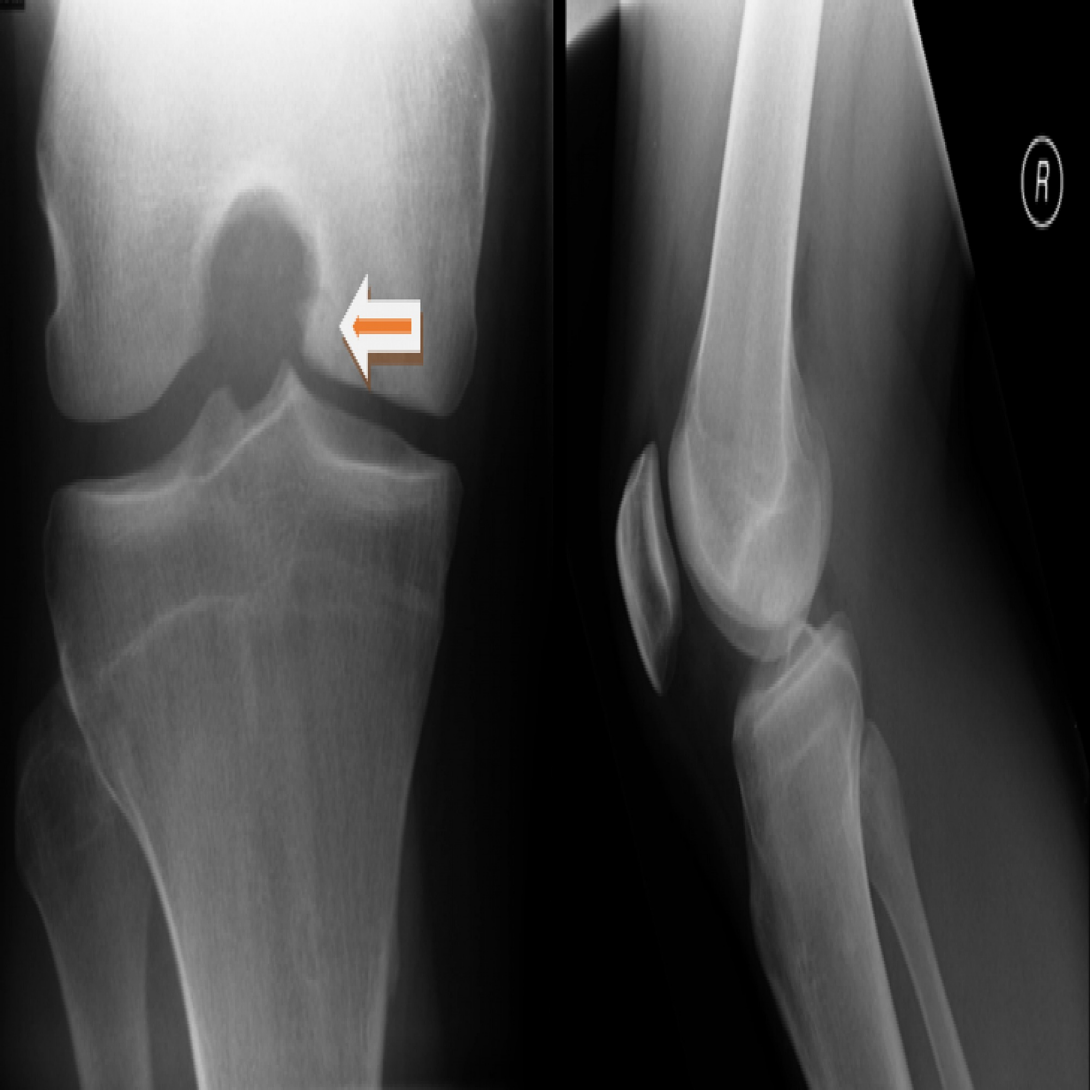
Radiograph showing Osteochondral lesion

**Figure 2:**
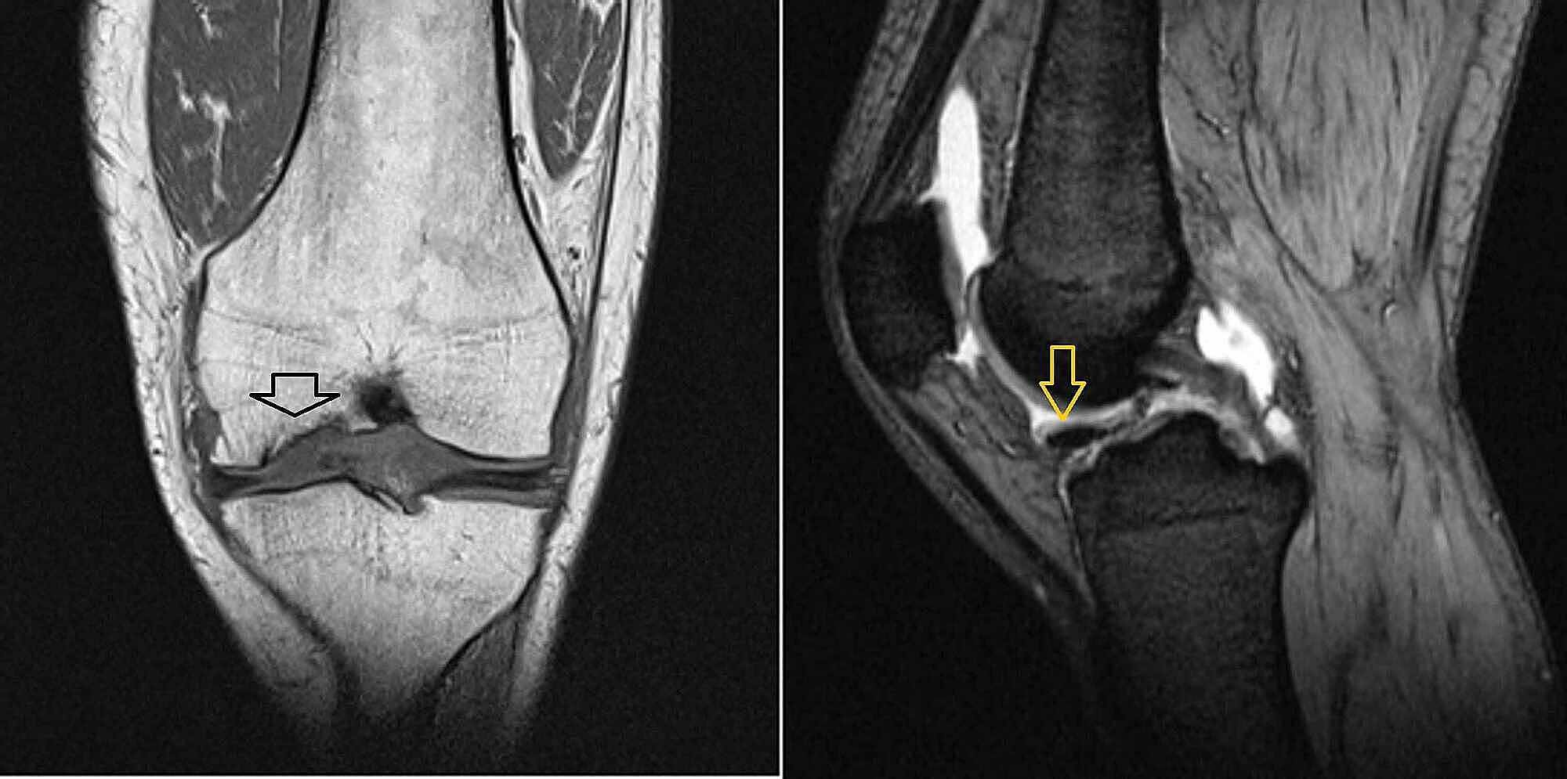
MRI scan (T1 and T2 weighted) revealing osteochondral defect on the weight-bearing surface of the medial femoral condyle and loose body in the anterior knee compartment

A 37-year-old man presented with persistent left knee pain of about six months’ duration. Concomitant symptoms included swelling, several episodes of locking and clicking, and a sense of instability especially in walking downstairs. Radiograph show an osteochondral lesion involving the medial femoral condyle (Figure 1). An MRI scan (Figure 2) revealed an unstable osteochondral lesion about 2 cm in diameter involving the medial femoral condyle and associated with a calcified detached fragment which was noted anteriorly within the joint extending towards the infrapatellar fat pad.

**Figure 3:**
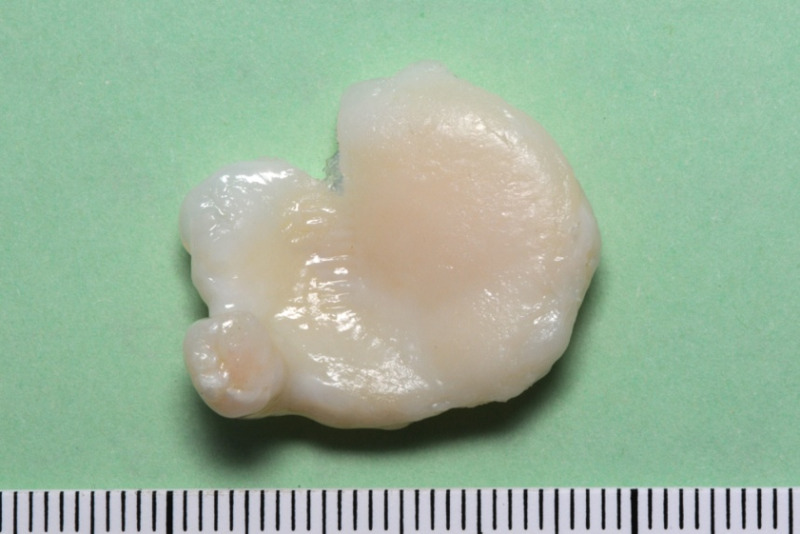
Loose osteochondral fragment removed from the anterior part of the knee joint

The patient underwent knee arthroscopy with removal of the loose osteochondral body (Figure 3), as it was not a fresh injury and therefore the fragment could not be fixed securely back to its original bed onto the femoral articular surface.

**Figure 4:**
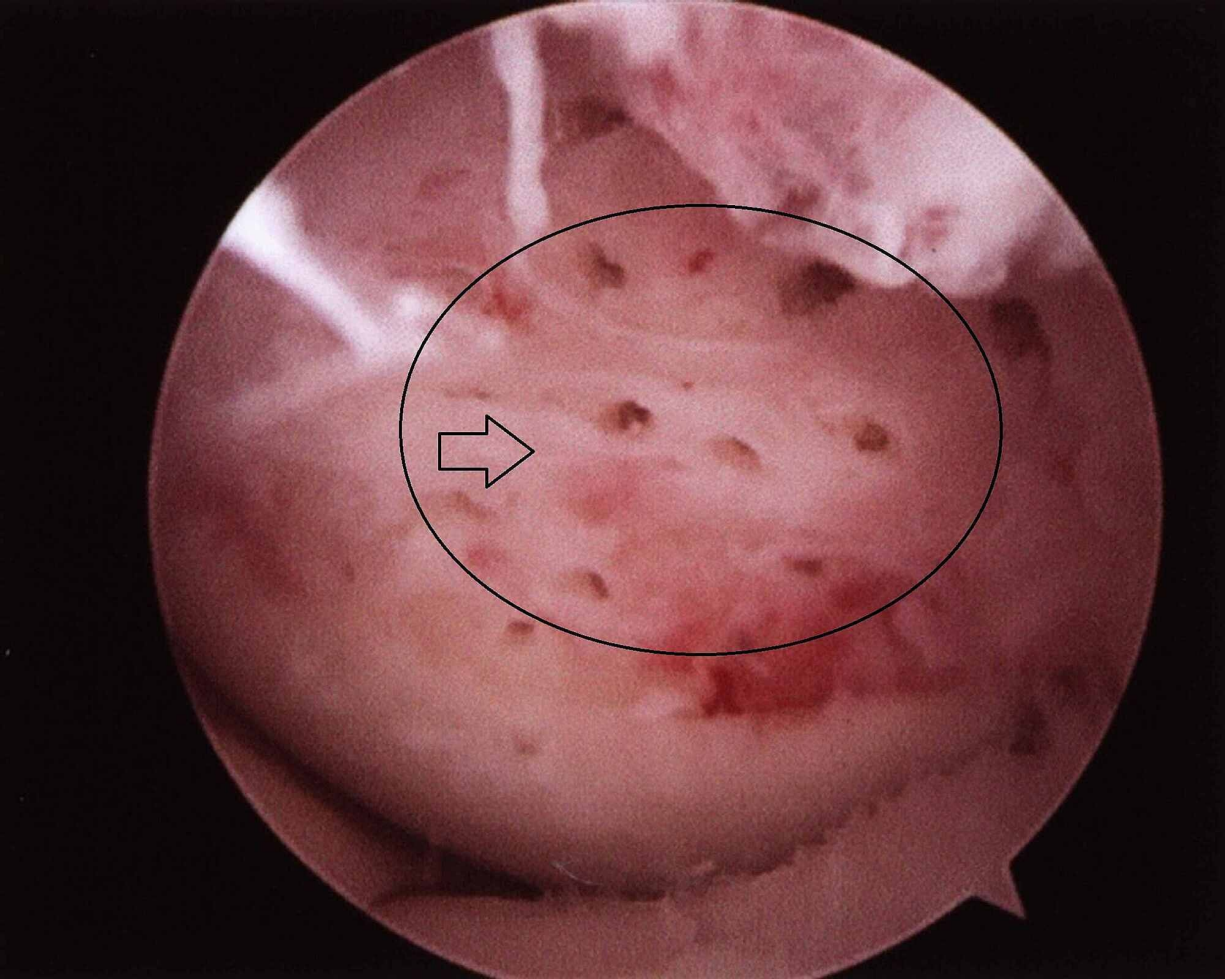
Microfracturing of the chondral defect

Following removal of the fragment, the defect was debrided with removal of the unstable cartilaginous remnants on the subchondral bone with an arthroscopic curette and shaver. Debridement was continued until steep and intact surrounding cartilaginous tissue was obtained. Removal of the calcified cartilaginous layer and achievement of a steep wall adhering closely to the subchondral bone are very important to establish an intact surface for the attachment of fibrous tissue. Finally, the lesion was micro fractured with a special hand awl creating several small holes 4-5 mm apart and to a depth of 3-4 mm in the subchondral bone (Figure 4).

**Figure 5:**
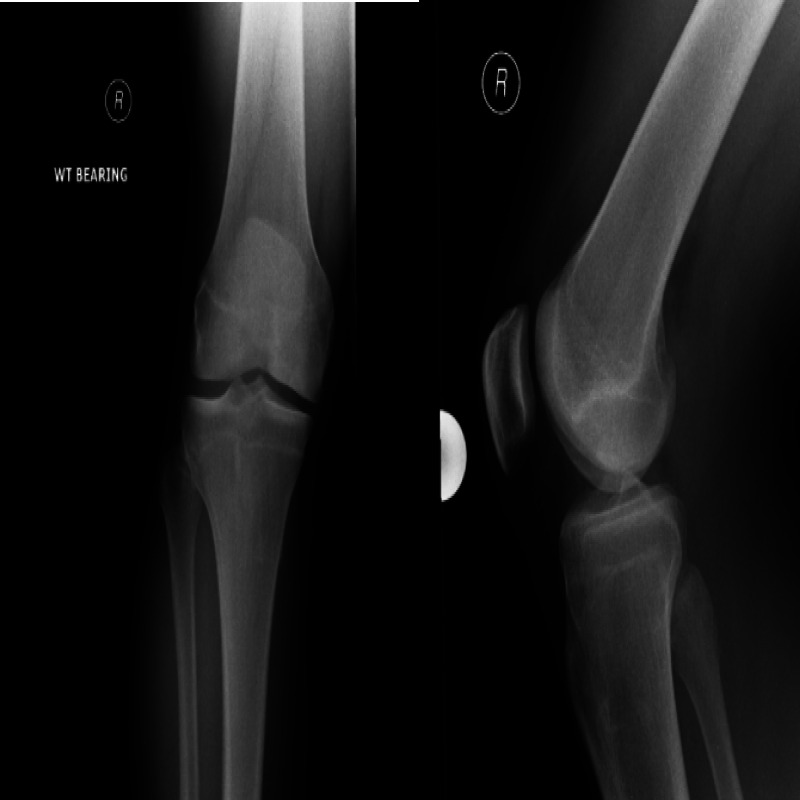
Post operative radiograph of knee

Postoperatively, the patient was treated with non-weight bearing for six weeks along with range of motion (ROM) exercises of his knee and quadriceps strengthening exercises (Figure 5).

The patient was under physiotherapist rehabilitation with progressive strengthening and loading at six to 12 weeks and neuromuscular retraining at 12-24 weeks. After six months following surgery, he has regained full range of motion (Western Ontario and McMaster Universities Osteoarthritis [WOMAC} index 56.2 preoperatively to WOMAC 7.2 postoperatively) and satisfactory muscle power and has returned to his previous activities.

## Discussion

For the management of full-thickness articular cartilage lesions of the knee, many treatment modalities have been defined. Hypoactive patients with few symptoms and incidental chondral lesions less than 2 to 3 cm^2^ should be managed with palliative methods such as debridement and lavage. In younger and more active patients with cartilaginous lesions measuring 1 to 5 cm^2^, techniques stimulating subchondral bone tissue have been used [10]. Currently, among techniques stimulating subchondral bone, the most popular is the microfracture technique [11]. Drilling of the subchondral bone leads to ingress of pluripotential mesenchymal stem cells, which arise from the vascular system, and growth factors into the defective region, and thus adherence to the surface of the bone is achieved [8,11]. Bleeding occurring after the procedure leads to the formation of a hematoma, which fills the defective region. Hematoma forms a fibrin plug in the area of the defect. The development of the fibrovascular repair tissue (i.e., granulation tissue) ensues [8,11].

There are several studies that show favorable results following microfracturing of the chondral defect in the weight-bearing surface of the femoral condyles. In 2003 Steadman et al. [8] used the microfracture technique in 71 patients aged under 45 years with traumatic chondral defects without any concomitant lesion and found increases in mean Lysholm scores (from 59 to 89 points) and mean Tegner scores (from 3 to 6 points) after approximately 11 years of follow-up.

In a randomized controlled trial comparing a group undergoing microfracture and a group undergoing autologous chondrocyte transplantation, Knutsen et al. [12] found better clinical results in younger and more active patients (aged <30 years) in both groups (P <0.007); although both methods had an acceptable short term clinical results. In another study by Pipino and co-workers, 46 patients with grade III and IV knee chondropathies, were consecutively treated with microfractures followed by application of hydrogel in the lesion site. They reported improvement in WOMAC score of 58.6 ± 11.0 in the study group to 7.1 ± 9.2 at six months and 2.9 ± 5.9 at 24 months. They concluded that microfracture “augmentation” might improve the clinical outcomes in osteochondral defects [13]. 

The clinical results after microfracture of Clanton and Delee grade III osteochondral lesions in the knee are age-dependent. A deterioration begins 18 months after surgery and is significantly pronounced in patients aged older than 40 years [14]. Younger patients with defects on the femoral condyles have the best long-term results.

## Conclusions

In conclusion, microfracture technique is quite effective with regard to the improvement of daily activities with a favorable impact on pain relief and overall satisfactory functional results. Furthermore, there seems to be a correlation between functional results and age, size of defect, location of defect, and BMI as prognostic parameters.
